# Making nursing a better place: a discrete choice experiment to elicit the job preferences of early-career nurses in England

**DOI:** 10.1136/bmjopen-2026-118717

**Published:** 2026-09-25

**Authors:** Ourega-Zoé Ejebu, Jane Ball, Mesfin G Genie, Julia Philippou, Joanne Turnbull

**Affiliations:** 1 School of Health Sciences, University of Southampton, Southampton, UK; 2 Royal College of Nursing, London, UK; 3 Newcastle Business School, The University of Newcastle Australia, Newcastle, New South Wales, Australia; 4 Florence Nightingale Faculty of Nursing, Midwifery & Palliative Care, King’s College London, London, UK

**Keywords:** Nursing research, Nurses, HEALTH ECONOMICS

## Abstract

**Background:**

In England, disproportionate numbers of registered nurses leave the National Health Service (NHS) early in their careers. Retaining early-career nurses could ease supply challenges. Most evidence—largely from outside the UK—indicates that retention factors among early-career nurses are within their employers’ control. Research is needed to understand the job preferences of early-career NHS nurses to inform and develop tailored retention policies.

**Objectives:**

To identify the job preferences of early-career NHS nurses and estimate the potential trade-offs between these job characteristics.

**Design and methods:**

A cross-sectional discrete choice experiment (DCE) administered through an online survey distributed by the Nursing and Midwifery Council. A DCE is a quantitative stated-preference method that presents respondents with hypothetical job scenarios with varying attributes and levels. Nurses chose their preferred job package from each set of alternatives, enabling the estimation of their job preferences and trade-offs. Following established practice, we employed qualitative work, including a literature review, focus groups and consultation with academic experts and stakeholders, which was undertaken before the DCE to inform the selection of attributes and levels. Quantitative findings are reported separately. We specified a mixed logit model to estimate the job preferences of early-career NHS nurses and explore preference heterogeneity.

**Setting:**

England, NHS.

**Participants:**

11 early-career nurses participated in the qualitative focus groups that informed DCE development, and 5696 early-career nurses were included in the final DCE analysis.

**Primary and secondary outcome measures:**

The DCE included eight job attributes: career development, organisational support, teamwork, management, personalised working, work pressure, recognition and salary. Salary was included as a continuous attribute to estimate the amount of annual salary respondents would be willing to forgo for improvements in the non-monetary job attributes.

**Results:**

Early-career NHS nurses preferred good teams with good collaboration,(b=1.178, p<0.01)supportive and accessible managers (b=0.817, p<0.01) and low work pressure $\begin{array}{l} \left(b=0.757,\;\;p<0.01\right) \end{array}$. They were willing to forgo £9419 in annual salary for good teamwork and collaboration, £6534 for supportive and accessible managers and £6055 for low-pressure work environments.

**Conclusions:**

Good teamwork and collaboration, supportive and accessible managers and low work pressure were highly valued by early-career NHS nurses. Healthcare managers should prioritise workplace environments that reflect these preferences as part of broader retention strategies.

**STRENGTHS AND LIMITATIONS OF THIS STUDY**

- This paper is the first discrete choice experiment focusing on early-career National Health Service (NHS) nurses to elicit their job preferences.
- The large sample size is representative of the nurse population and was drawn from the Nursing and Midwifery Council—the sole independent regulator for nurses (and midwives) in the UK—making it a more comprehensive source of data than a professional body, trade unions or set of employers and rendering our results more generalisable.
- We used published literature, qualitative data collection, academic experts and stakeholder consultation to identify the relevant discrete choice experiment job attributes and levels.
- Nurses who have left the NHS were not included in the study.

## Introduction

The early departure of newly qualified nurses—those with 5 or fewer years of experience—exacerbates nursing shortages in many countries, and England is no exception. In the National Health Service (NHS), early-career nurses exhibit the highest leavers’ rates in comparison to other groups.^1–3^ The departure of these nurses further strains the labour market. The loss of recently qualified nurses creates a gap in the year that they graduate, as well as a potential loss of service in healthcare in the years to come. Shortages of nurses compromise the quality of care, as nurses experience increased workloads, and patients may experience missed care.^4^ As a result, remaining staff are more likely to experience burnout, sickness absences, low morale and are more likely to intend to leave their jobs and/or the profession.^5–7^ The most recent and global strike actions have highlighted that the nursing workforce expects significant improvements in their working conditions (and salary) to deliver the standard of care they aspire to.^8^

There is a vast literature on the workforce push-pull factors among early-career nurses.^9–12^ Findings indicate that for these nurses, retention is positively associated with job satisfaction,^10 12–15^ organisational support such as preceptorship and/or mentoring,^9^ supportive leadership, opportunities for career advancement, team support, work–life balance and decision-making in patient care.^11^ Such findings shed light on what could retain early-career nurses in the profession, but these are largely drawn from the international literature (the USA, Canada, Australia and South Korea). This may limit the application of findings to England, and the NHS in particular. This direct applicability of international findings may be limited due to contextual differences and the uniqueness of the NHS in terms of structure, including nurses’ career paths. Furthermore, and crucially, the relative importance of job factors associated with retaining early-career nurses is not known.

Interventions (eg, NHS People Promise^16^) and policies (eg, N50k Nurse Programme^17^) have been implemented to boost nurse retention by focusing on flexible working patterns, enhancing work culture and leadership across organisations. Yet, it is unclear which of these interventions are most important, and whether they are valued equally across nurses at different career stages. A better understanding of early-career nurses’ job preferences would enable targeted retention strategies to be developed that reflect the most highly valued job attributes.

This study aimed to address this knowledge gap by undertaking a discrete choice experiment (DCE) to elicit the job preferences (ie, what they value most) of early-career NHS nurses and estimate the potential trade-offs between different job characteristics. A DCE is a well-established economic method, which finds its roots in Lancaster’s economic theory of value and McFadden’s extended work on random utility theory.^18–20^ These theories stipulate that individuals derive utility (ie, satisfaction) not from a good (here, a hypothetical job on offer), but from its attributes and levels. In a DCE, participants face a series of hypothetical job scenarios with different attributes (eg, characteristics) and levels. Results from the DCE are beneficial in identifying and quantifying the most and least important attributes. In addition, we can estimate the trade-offs made between these characteristics to know how to compensate nurses for the least favourable job characteristics, or how much they are willing to pay (sacrifice) for the most favourable job characteristics.

DCEs are increasingly used in health services research to elicit the preferences of health professionals.^21–28^ A few studies have used DCE to explore nurses’ job preferences for working in rural areas, and most were conducted in low- and middle-income countries.^29–34^ To date, only a few DCE studies exploring the job preferences of nurses, midwives (and doctors) have been conducted in high-income countries.^22 23 25 35^ In England, to our knowledge, no DCE has been conducted to inform about the job preferences of early-career NHS nurses.^21^

## Methods

### Study design: qualitative work informing the DCE

Our study was conducted in two stages. In stage 1, we aimed to identify and select the DCE attributes and levels. Following best practice,^36–38^ we conducted a qualitative study to explore in detail what early-career NHS nurses value about their profession, what they expect from their employers/workplaces to stay in their jobs and the nursing profession, and explore push-pull factors associated with their decision to remain in or leave their jobs and/or nursing.

Online focus groups and interviews were conducted with 11 early-career nurses working in NHS England. Participants were recruited via several channels, including social media and an earlier survey conducted in 2023, where respondents could agree to be contacted for future research.^39^ We purposively sampled nurses to represent different demographic characteristics and job roles and capture a diversity of experiences and views across participants. Recruitment was conducted until reaching data saturation, that is, until we were confident that we had collected sufficient data to yield enough information power to elucidate the aims of the study.^40^ More details on the qualitative study findings can be found elsewhere.^41^ We also searched the literature,^42–44^ as well as consulted with experts’ opinions, members from our nurse and patient involvement group and key stakeholders (including representatives from the Department of Health and Social Care, DHSC and NHS England, NHSE).

A thematic analysis of the qualitative data and discussion with experts and stakeholders identified eight job attributes, each with different levels (table 1). Career development (career opportunities, education and professional development) was included because early-career nurses have clear expectations for career growth, to progress and avoid stagnation. Other studies also found a positive association between career advancement and retention.^45 46^ We included organisational support (support from workplace) as it is considered an important retention factor for early-career nurses.^42 44 47^ This support, when available, tends to be offered to help early-career nurses when they transition from student to nurse. For instance, early-career nurses may be offered preceptorship and/or mentoring programmes, which may strengthen their confidence, reduce their feelings of inadequacy and enhance retention.^48^

**Table 1:** List of attributes and levels to explore the job preferences of early-career NHS nurses

Definition of job characteristics	Description of levels
Career development: career opportunities, education and professional development	Limited opportunities for career development Good opportunities for career development
Organisational support: support (preceptorship, mentoring) from workplace	Lack of organisational support, poorly structured Good organisational support, well structured
Teamwork: collaboration and effectiveness of teamwork	Poor collaboration and teamwork Good collaboration and teamwork
Management: accessibility and support from management	Unsupportive and inaccessible Supportive and accessible
Personalised working: flexible working patterns to support work–life balance	Lack of flexible working patterns Good flexible working patterns
Work pressure: workload and intensity associated with the role	High work pressure Low work pressure
Recognition: experience and skills are recognised and valued by colleagues	Low recognition, skills not valued Good recognition, skills are valued
Salary: variation in income	Same income 10% increase in income 25% increase in income

NHS, National Health Service.

Teamwork (collaboration and effectiveness of teamwork) was considered an important factor given the numerous tasks that require nurses to work with other colleagues. Positive relationships with colleagues are particularly key for early-career nurses, as they may act as a buffer in a challenging and demanding work environment.^12^ Similarly, management (accessibility and support from management) was included, considering the influential role of managers in several job outcomes, including job satisfaction and retention.^49 50^ Managers who are accessible and supportive are particularly valued by early-career nurses to enhance working conditions.^51^ Personalised working (flexible working patterns to support work–life balance) was included. While flexibility in schedules/rostering may be complex to achieve, given patient care requires continuous provision, early-career nurses desire to have work schedules enabling them to maintain a healthy work–life balance. This may be attributed to the fact that nursing is a female-dominated profession^14 52^ and early-career nurses seem to value schedule flexibility, enabling them to better handle caring responsibilities (eg, children, elderly family members).^41 53^

Work pressure (workload and intensity associated with the role) was included, as early-career nurses work in demanding work environments. They may not have the required resources to care for patients to the standard they aspire to. For instance, the lack of staff and/or equipment may exacerbate their workload and working conditions. The literature suggests that workload is associated with intention to leave.^54 55^ Recognition (experience and skills are recognised and valued by colleagues) was included as early-career nurses desire to be recognised and valued by their colleagues and the leadership team.^56 57^ In contrast, the lack of recognition may adversely influence nurses’ job satisfaction and/or the decision to stay in their jobs. Finally, salary (variation in income) was included as income is key to enabling early-career nurses to face the costs of living,^58^ as well as reflect their skills and experience. It was also included to calculate ‘willingness-to-pay’ for changes in all other job characteristics. The salary levels were chosen after discussion with early-career nurses, experts and reviewing the literature.^22 25 26^ Percentage was chosen—rather than pay scale—considering the regional income variation, as well as to avoid unrealistic incomes for respondents working part-time. Table 1 displays the list of all the DCE attributes, their definitions and levels.

### Study design: designing the DCE

In stage 2, we design the cross-sectional DCE. Specifically, we used the Ngene experimental design software^59^ to generate a D-efficient design (D-efficient level=0.45) with fixed priors.^60^ Priors were determined based on the literature review and aforementioned findings from the qualitative analysis. We selected a fractional factorial design based on the eight attributes—seven attributes with two levels and one attribute with three levels (2^7^×3^1^)—to generate 12 job choices. To reduce the cognitive burden among respondents, they were blocked into two versions, that is, each respondent received a random version with six job choices. We checked the DCE designs to ensure they met the required criteria, including orthogonality, level balance with minimum overlap and utility balance.^61 62^ The orthogonality in the DCE attributes was observed during both the DCE design and preregression analyses, with Pearson correlation coefficients lower than 0.35^37^ (online supplemental table 7 appendix 1). Finally, we used Louviere’s formula to calculate the required sample size for a DCE. It is as follows: $\left(500\frac{L^{max}}{JS}\right)$. The formula indicated that a minimum sample size of 125 would enable us to estimate the coefficients from our regressions.^63^ Here $L^{max}$ is the largest number of levels for any of the attributes, J is the number of alternatives per choice task, and S is the number of choice sets per respondent.^63^ As Louviere’s formula is a heuristic sample-size guideline rather than a power calculation, an explicit level of precision cannot be inferred. Therefore, the study’s precision is obtained from the estimated SEs and CIs of the regression models.^64^

The DCE was then incorporated into a more comprehensive questionnaire created with an online survey platform, Qualtrics.^65 66^ The questionnaire explored the working conditions of early-career nurses, their experiences of joining the profession and demographic-related information. The survey was completed by nurses working across all settings (including the non-NHS sector). However, only nurses working in the NHS completed the DCE section because the study was part of a larger project (ie, N50k Evaluation Study) aimed at evaluating the Government’s plan to increase the NHS nursing workforce and improve retention (ie, N50K Nurses Programme).^17 67 68^ Findings from the N50K Evaluation Study suggest that retention strategies developed under the N50K Programme should extend beyond workforce growth targets and instead prioritise improvements to workplace culture, leadership accessibility and support systems, which are shown to be more influential in retaining early-career nurses.

In a section that explored early-career nurses’ job preferences, respondents were asked, “If you were offered these two nursing jobs, which job would you choose: Job A or Job B?” An ‘opt-out’ option was not included in the DCE design, as we captured intention to stay in the same job with the same/different employer in another section of the survey. We included an example job alternative with Best (Job A) and Worst (Job B) levels to check whether respondents answered rationally, as well as to familiarise themselves with the tasks. The majority of respondents (98.2%) answered rationally to the example question, suggesting they understood the DCE choice tasks. Figure 1 (see online supplemental appendix 2) shows a real example of the job choice nurses faced in the survey.

**Figure 1:**
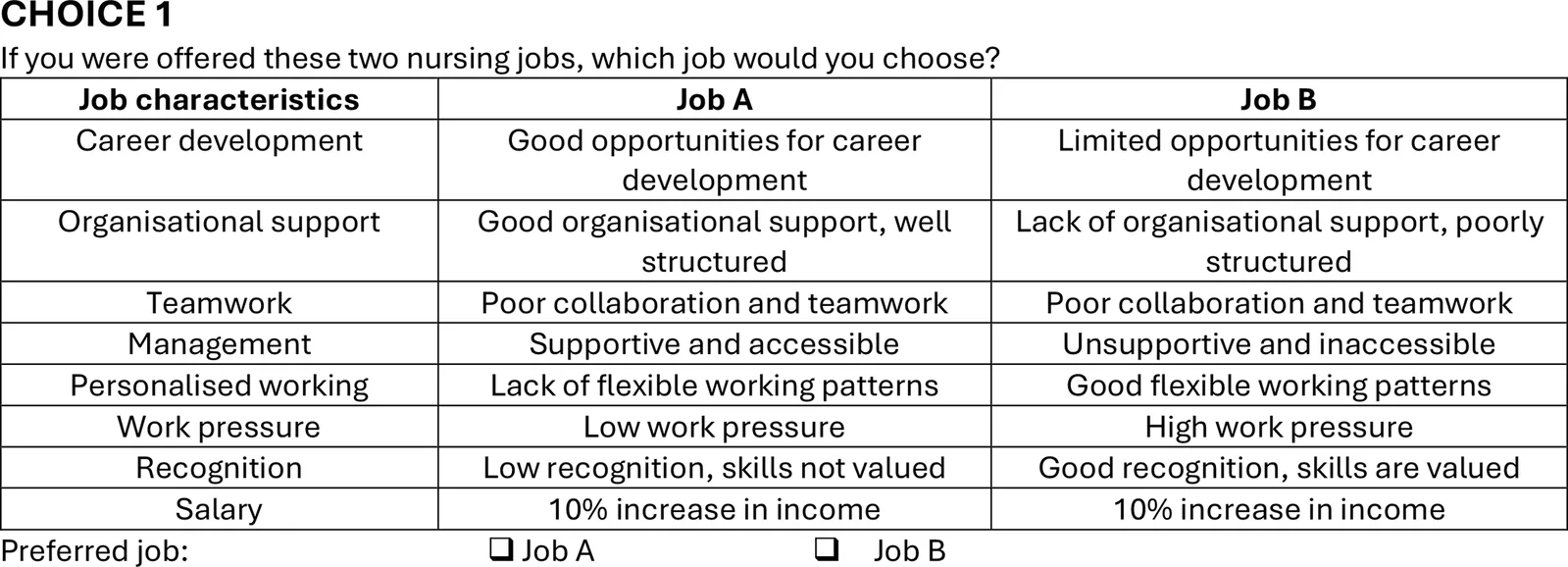
Example of the DCE job choice to explore job preferences among early-career NHS nurses.

### Recruitment of participants

Before the final online launch and following established practice,^37 69^ the survey—including the DCE—was piloted in ‘think-aloud’ sessions led by the research team. Five early-career nurses provided feedback about the survey format, length and content, and about the DCE specifically. Feedback related mainly to some terminology and language used in the survey, which were amended by the authors. No comments were made about the terminology and definition of the DCE attributes and their levels.

The survey was administered online by the Nursing and Midwifery Council (NMC) on behalf of the research team. The survey/DCE was piloted with a random sample of early-career nurses (N=800) to verify its consistency and conduct preliminary analyses. Having obtained satisfactory pilot results, no further changes were made. Since the study was part of a larger project (ie, the N50K Nurses Programme), the survey was sent to all nurses who first registered with the NMC between 2019 and 2024 and were working in England at the time of the study (N=143 133). This also includes internationally-educated nurses (IENs) who were recruited in large numbers as part of the N50k programme.^70 71^ All nurses in the sample had less than 5 years of work experience in the UK but may have included nurses with prior work experience in other countries.

The survey was open from May to July 2024, and one reminder was sent by the NMC. To encourage participation, we indicated that for every survey response, early-career nurses could choose that a donation be made on their behalf (£0.50) to the following charity: Marie Curie, Médecins Sans Frontières (MSF) or Cavell. Respondents could also choose that no donation be made on their behalf. While rarely used in UK surveys, we perceived that the use of a charitable incentive could promote social impact and enable respondents to give back while encouraging participation in the survey.^72^

### Analysis

We used the random utility maximisation framework.^19^ In this model, it is assumed that in each choice task (t=1,…,T), early-career nurses (n=1,…,N) derive utility (satisfaction) $U_{njt}$ for a job (j=1,…,J). Then, they choose to accept the job if the associated utility exceeds the utility of the other job on offer. The utility of the given job (ie, dependent variable) is a combination of the deterministic (ie, observables) and random (unobservable) components. The latter is captured by the error term, which is independently and identically distributed.

A mixed logit model was applied to estimate the job preferences of early-career NHS nurses, quantify trade-offs between job characteristics and examine preference heterogeneity. The mean coefficients indicate the relative direction and strength of preferences for the attribute levels. Statistically significant SDs of the random parameters indicate heterogeneity in preferences across respondents.^73^ Except for salary, which was specified as a fixed, continuous variable, the non-monetary job attributes were specified as random parameters. The Worst (least favourable) level of each attribute served as the reference category. We used 1000 Halton draws to improve simulation accuracy. The utility specification was as follows:


$$\begin{array}{l} U_{njt}=\beta_{0}Worst_{ntj}+\beta_{1}Career\_Development_{ntj}+\\ \beta_{2}Organisational\_Support_{ntj}+\beta_{3}Teamwork_{ntj}+\\ \beta_{4}Management_{ntj}+\beta_{5}Personalised\_Work_{ntj}+\\ \beta_{6}Work\_Pressure_{ntj}+\beta_{7}Recognition_{ntj}+\beta_{8}Salary_{ntj} \end{array}$$

Marginal willingness to pay (WTP) was estimated to express preferences for the non-monetary job attributes in salary terms. Because salary was coded as a percentage increase and its coefficient was positive, the compensating variation for an improvement in a non-monetary attribute was calculated as the negative ratio of the attribute coefficient to the salary coefficient. The resulting negative percentage represents the reduction in salary respondents would be willing to accept, or equivalently, the salary they would be willing to forgo, to obtain the more favourable attribute level.^22 25 74^ It should therefore not be interpreted as willingness to accept compensation for an undesirable job characteristic, although reversing the direction of the comparison would yield an equivalent compensating amount under the linear specification. Percentage estimates were converted to annual monetary values by multiplying their absolute magnitude by the mean annual income of the estimation sample.

Two sensitivity analyses were conducted to verify the robustness of our results. Specifically, job preferences were explored by region (East of England, London, Midlands, North East and Yorkshire, North West, South East and South West) and healthcare sector. The healthcare sector was regrouped into two main categories—Primary and Secondary Care sectors—using the respondents’ workplace and field-of-practice information.

We also explored the job preferences and potential trade-offs for IENs, specifically in Nigeria, Ghana, India and the Philippines, regrouped in the same model, and compared the results with those of nurses who first registered in the UK. These countries are of particular interest because the majority of the international nurses recruited as part of the N50K Nurses Programme originated from these countries. Indeed, England reached its 50 000 nurse target by recruiting 43 000 IENs (86%), as part of the N50k Nurse Programme.^70^ Understanding the preferences of IENs is key to retaining this valuable and recently hired workforce, ensuring that interventions target what they value the most, the extent to which they should be (financially) compensated for the least-favourable job characteristics or what they would forgo for the most-favourable job characteristics.

We conducted all the analyses with Stata V.19^75^ and used 1% (p<0.01) and 5% (p<0.05) as the significance levels.

## Results

### Sample description

Of the 143 133 early-career nurses invited to participate, 10 192 clicked on the survey. Respondents were excluded if they were no longer on the NMC register (N=1), employed outside of nursing (N=73), not in paid employment (N=99) or not living in England (N=17). In addition, we excluded respondents who did not record their employment situation (N=647) and had mainly missing values (N=716). This yielded a sample size of 8639 (6% response rate). Among these, 5696 respondents were included in the final analysis as they met the inclusion criteria (ie, working in NHS England) and fully completed all six DCE job choices.

On average, respondents were 33.5 years of age (SD: 5.78) and female (83.3%), which is consistent with NMC data on newly-registered nurses.^76^ The majority held a nursing degree (80%) and had an adult registration (83.2%). Half (50.4%) of the respondents first registered in the UK, 31.9% first registered in Asia (predominantly Philippines and India—93.7% in total), and 14.4% in Africa and the Caribbean Islands (predominantly Ghana and Nigeria—65.3% in total), reflecting the patterns of the N50k international recruitment initiative.^70^ In our data, there is a slight oversampling of IENs when compared with the NMC data of newly registered, which revealed that in 2022, 40% of all new nurses registered came from low- and middle-income countries.^77^ 36.5% of the respondents were White, 35.9% were Asian/British, and 21.2% were Black/British. Consistent with newly-registered nurses starting at the lowest band in the UK,^78^ 77% of the respondents were Band 5 nurses (ie, earning the equivalent of £28 407–£34 581 at the time of the study). Overall, 84.2% perceived that their salary was not appropriate to their skills and expertise. The mean length of work experience (since first registration) was 8.0 years (SD: 7.0), although all nurses had less than 5 years of work experience in the UK (table 2).

**Table 2:** Characteristics of early-career NHS nurses (N=5696)

First NMC registration	Freq.	%	Qualification		
2019	870	15.3	Nursing degree	4557	80.0
2020	839	14.7	Nursing diploma	822	14.5
2021	1179	20.7	Masters	276	4.9
2022	1139	20.0	Other	8	0.1
2023	1313	23.1	PhD	4	0.1
2024	352	6.2	Missing	29	0.5
Missing	4	0.1	Registration		
Gender			Adult	4740	83.2
Female	4742	83.3	Mental health	621	10.9
Male	850	14.9	Children	271	4.8
PNTS	104	1.8	Learning disabilities	63	1.1
Ethnic group			Other	1	0.0
White	2077	36.5	Place of first registration		
Asian	2042	35.9	UK	2868	50.4
Black	1209	21.2	Asia	1818	31.9
PNTS	217	3.8	Africa and Caribbean Islands	821	14.4
Mixed	112	2.0	Rest of the world	110	1.9
Missing	39	0.7	Europe	79	1.4
Children			Employment situation		
Yes	3127	54.9	Employed	5686	99.9
No	2551	44.8	Self-employed	10	0.2
Missing	18	0.3	Current employer		
Location			NHS	5578	97.9
Midlands	1065	18.7	GP practice	118	2.1
South East	867	15.2	Field of practice		
North West	816	14.3	Adult general	2233	39.2
London	794	13.9	Adult critical care	831	14.6
North East and Yorkshire	751	13.2	Mental health	579	10.2
South West	730	12.8	Other acute specialties	438	7.7
East of England	609	10.7	Community care	438	7.6
Other	64	1.1	Primary care	358	6.4
Pay band			Children and young people	231	4.1
5 (£28 407–£34 581)	4403	77.3	Older people’s care	163	2.9
6 (£35 392–£42 618)	1133	19.9	Paediatric critical care	123	2.2
7+ (£43 742–£68 525)	156.0	2.8	Rehabilitation	95	1.7
Missing	4	0.1	Several specialties	61	1.1
Appropriateness of pay			Research and Education	67	1.2
No	4797	84.2	Learning disabilities	39	0.7
Yes	494	8.7	Palliative care	20	0.4
Don’t know	320	5.6	Other	16	0.3
Missing	85	1.5	Missing	4	0.1
Age and years of experience (mean)			Work patterns		
Age (SD): 33.5 (5.8) (Min–Max: 21–46)			Full-time	5035	88.4
Experience (SD): 8.0 (7.0) (Min–Max: 0–40)			Part-time	484	8.5
			Missing	129	2.3
			Occasional hours	48	0.8

GP, general practitioner; NHS, National Health Service; NMC, Nursing and Midwifery Council; PNTS, prefer not to say.

### Mixed logit model results: full sample

Table 3 presents the mixed logit estimates, with the Worst (or least favourable level) used as the reference category for each non-monetary attribute. The statistically significant alternative-specific constant indicates a left-position bias: Job A was selected in 54.9% of choices, compared with 45.1% for Job B. All eight attributes were significantly associated with choices, and the signs of the coefficients were consistent with a priori expectations, supporting the face validity of the results.

**Table 3:** Mixed logit results of the job preferences among early-career NHS nurses

Job characteristics	Coefficients (SEs)	SD (SEs)
Good collaboration and teamwork	1.178***	0.016
(Ref: Poor collaboration and teamwork)	(0.034)	(0.256)
Supportive and accessible managers	0.817***	0.005
(Ref: Unsupportive and inaccessible managers)	(0.021)	(0.140)
Low work pressure	0.757***	0.509***
(Ref: High work pressure)	(0.025)	(0.033)
Good opportunities for career development	0.698***	0.461***
(Ref: Limited opportunities for career development)	(0.019)	(0.034)
Flexible working patterns	0.575***	0.224***
(Ref: Lack of flexible working patterns)	(0.017)	(0.059)
Good recognition, skills are valued	0.566***	0.197***
(Ref: Low recognition, skills are not valued)	(0.017)	(0.068)
Good organisational support, well structured	0.460***	0.367***
(Ref: Lack of organisational support, poorly structured)	(0.018)	(0.040)
Salary	0.042***	Fixed
	(0.001)	
Constant (Job Choice A)	0.193***	0.000
	(0.015)	(0.058)
Observations	68 352	
Number of respondents	5696	
Log-likelihood	−17257.6	

* *p<0.05, ***p<0.01.

NHS, National Health Service.

Results suggest that early-career NHS nurses preferred good teamwork and collaboration (b=1.178,p§amp;lt;0.01), supportive and accessible managers (b=0.817,p§amp;lt;0.01), low work pressure (b=0.757,p§amp;lt;0.01) and good opportunities for career development (b=0.698,p§amp;lt;0.01). This was followed by a preference for flexible working patterns (b=0.575,p§amp;lt;0.01), being recognised and valued (b=0.566,p§amp;lt;0.01) and lastly, good opportunities for organisational support that is well structured (b=0.460,p§amp;lt;0.01). As expected, early-career nurses preferred a higher percentage of income change (b=0.042,p§amp;lt;0.01). Finally, estimates reveal that there is heterogeneity of preferences for all job characteristics—except for teamwork and supportive managers—as indicated by the statistical significance of the SD coefficients. This suggests that early-career NHS nurses value teamwork and managers more or less equally, in comparison to the other job attributes.

Notably, results from sensitivity analyses (ie, region and healthcare sector) also align with the main models. They reveal that, across all specifications (1) all job attributes are significant and of the expected signs and (2) good collaboration and teamwork, supportive management and low work pressure are among the top three job attributes preferred by early-career NHS nurses (Results in online supplemental appendix 3 and 4).

Table 4 reports marginal WTP estimates as the percentage of annual salary respondents would be willing to forgo for an improvement from the reference level to the favourable level of each attribute, together with the corresponding annual monetary amount.

**Table 4:** Willingness to pay (WTP), expressed as the annual salary respondents would be willing to forgo for favourable job attributes

Job characteristics	WTP (%) (ll-ul)	Annual average income (£)
Good collaboration and teamwork	−28.2	9418.7
	(−26.6 −17.6)	
Supportive and accessible managers	−19.5	6534.4
	(−20.5 −18.6)	
Low work pressure	−18.1	6055.2
	(−19.3 −17.0)	
Good opportunities for career development	−16.7	5579.7
	(−17.6 −15.8)	
Flexible working patterns	−13.8	4597.8
	(−14.6 −13.0)	
Good recognition	−13.5	4527.5
	(−14.3 −12.7)	
Good organisational support	−11.0	3675.4
	(−11.8 −10.2)	

WTPs were obtained using the estimates from the mixed logit model with the Worst level as the references.

ll, lower limit; ul, upper limit.

Early-career NHS nurses were willing to forgo 28.2% of their annual salary for good teamwork and collaboration, equivalent to £9419. This represents the largest WTP in comparison to other attributes. To work with accessible and supportive managers, nurses would pay 19.5% of their annual income, or £6534—the second largest WTP. This is followed closely by working in a low-pressure environment, where 18.1% of nurses’ annual income would be forgone, the equivalent of £6055. The corresponding estimates were 16.7% (£5580) for good career-development opportunities, 13.8% (£4598) for flexible working patterns, 13.5% (£4528) for recognition, and 11.0% (£3675) for well-structured organisational support. These estimates should be interpreted as marginal rates of substitution expressed in salary terms rather than as literal amounts that nurses would necessarily pay out of pocket.

### Mixed logit model results: subgroup analyses for UK and IENs nurses

Table 5 presents mixed logit estimates for nurses first registered in the UK and IENs from Nigeria, Ghana, India and the Philippines. The UK subgroup was included to compare the preferences of domestically and IENs. All UK-registered nurses in the sample had 5 years or less of nursing experience, whereas some IENs had more than 5 years of experience before registering in the UK.

**Table 5:** Results of mixed logit regressions to explore the job preferences of early-career NHS nurses: UK and IENs

Job characteristics	UK		IENs	
	Mean (SEs)	SD (SEs)	Mean (SEs)	SD (SEs)
Good collaboration and teamwork	1.393***	0.316*	0.872***	−0.005
(Ref: Poor collaboration and teamwork)	(0.058)	(0.183)	(0.045)	(0.110)
Supportive and accessible managers	0.959***	0.279***	0.647***	0.001
(Ref: Unsupportive and inaccessible managers)	(0.035)	(0.080)	(0.028)	(0.071)
Low work pressure	0.761***	0.572***	0.710***	0.360***
(Ref: High work pressure)	(0.039)	(0.055)	(0.033)	(0.057)
Good opportunities for career development	0.716***	0.517***	0.656***	0.444***
(Ref: Limited opportunities for career development)	(0.030)	(0.051)	(0.027)	(0.050)
Flexible working patterns	0.673***	0.333***	0.449***	0.132
(Ref: Lack of flexible working patterns)	(0.029)	(0.073)	(0.024)	(0.135)
Good recognition, skills are valued	0.653***	0.360***	0.456***	−0.002
(Ref: Low recognition, skills are not valued)	(0.028)	(0.089)	(0.024)	(0.106)
Good organisational support, well structured	0.467***	0.454***	0.446***	0.238***
(Ref: Lack of organisational support, poorly structured)	(0.028)	(0.056)	(0.026)	(0.081)
Salary	0.046***		0.037***	
	(0.001)		(0.001)	
Constant (Job choice A)	0.204***	0.003	0.171***	−0.001
	(0.024)	(0.079)	(0.023)	(0.097)
Observations	34 416		30 542	
Number of respondents	2868		2239	
Log-likelihood	−8541.2		−7143.7	

* p<0.10, **p<0.05, ***p<0.01.

IENs, internationally educated nurses; NHS, National Health Service.

Across the two models, the job attributes are all statistically significant and of the expected signs, indicating face validity of the results. This suggests that all the job attributes are important for early-career NHS nurses, and as expected, they prefer the best levels of each attribute in comparison to the worst levels. In contrast to UK early-career NHS nurses, most estimates for early-career NHS IENs show that there is no or little heterogeneity of preferences in the sample, as indicated by the absence of statistical significance of the SD’s coefficients. This may suggest that early-career NHS IENs value teamwork, supportive management, low work pressure and recognition more or less equally.

The four most highly valued attributes were similar across the two subgroups, although their ordering differed slightly. Good teamwork and collaboration, supportive and accessible managers, low work pressure and good career-development opportunities were among the most valued attributes for both UK-registered nurses and IENs. Good teamwork and collaboration ranked first in both groups. Supportive and accessible managers ranked second among UK-registered nurses, whereas low work pressure ranked second among IENs.

Using the subgroup-specific mixed logit estimates, we calculated marginal WTP as the proportion of annual salary respondents would be willing to forgo for each favourable attribute level. Larger absolute WTP estimates indicate stronger preferences, conditional on the salary coefficient. Table 6 presents these percentages and their corresponding annual monetary values (income equivalent (£)) for UK-registered nurses and IENs.

**Table 6:** Willingness to pay (WTP), expressed as annual salary respondents would be willing to forgo for favourable job attributes, by registration group

Job characteristics	UK		IENs	
	WTP (ll-ul)	Annual average income (£)	WTP (ll-ul)	Annual average income (£)
Good collaboration and teamwork	−30.5	£10 361.6	−23.56	£7728.8
	(−32.9 −28.0)		(−26.20 −20.92)	
Supportive and accessible managers	−21.0	£7134.7	−17.48	£5736.1
	(−22.5 −19.5)		(−19.26 −15.71)	
Low work pressure	−16.7	£5664.0	−19.19	£6296.0
	(−18.3 −15.0)		(−21.19 −17.19)	
Good opportunities for career development	−15.7	£5323.2	−17.72	£5812.2
	(−16.9 −14.4)		(−19.23 −16.20)	
Flexible working patterns	−14.7	£5003.5	−12.13	£3979.8
	(−15.9 −13.5)		(−13.44 −10.82)	
Good recognition	−14.3	£4857.7	−12.32	£4043.2
	(−15.5 −13.1)		(−13.64 −11.00)	
Good organisational support	−10.2	£3477.2	−12.04	£3951.4
	(−11.4 −9.0)		(−13.52 −10.57)	

WTPs were obtained using the estimates from the mixed logit model with the Worst level as the references.

IENs, internationally educated nurses; ll, lower limit; ul, upper limit.

## Discussion

To our knowledge, this is the first DCE exploring the job preferences of early-career NHS nurses. Results from our study show what nurses in this career stage value the most and where interventions should be prioritised or refocused to prevent them from leaving their role or the profession. Our study also contributes to understanding the extent to which they would be willing to make trade-offs among those job characteristics. Furthermore, the results were consistent when exploring job preferences by region and healthcare sector, and thus can provide robust, evidence-based outputs for health workforce leaders and decision-makers.

All job attributes were preferred by early-career NHS nurses, and notably, salary was not the most important. This may reflect the fact that nursing is often considered a vocational profession that attracts individuals who desire to care for and help people when they are particularly vulnerable.^79 80^ This finding also reinforces the notion that interventions solely targeting pecuniary factors are unlikely to resolve retention issues among early-career nurses. These findings offer clear direction for NHS policymakers and workforce planners. Strategies should prioritise improving team dynamics, supportive leadership and manageable workloads, areas identified as more highly valued by early-career NHS nurses. Notwithstanding, adequate salaries are important to nurses. In our sample, 84.2% of early-career NHS nurses indicated that their salary was not commensurate with their skills and experience, findings also reported in other literature.^11 81^ Similarly, our previous research found that some nurses consider that a starting salary at Band 6 (£35 392) rather than Band 5 (£28 407) would be a better reflection of their skills, experience and enable them to face increasing living costs.^41^

While all attributes were important, teamwork, accessible and supportive managers and low work pressure were among the most valued job factors, which is consistent with other studies.^81–83^ While these attributes may suggest that a large-scale culture change and reducing pressure across the NHS are important, these may be difficult to achieve. Whether the team relates to immediate or indirect colleagues is difficult to discern from the results. However, given the considerable number of tasks that are executed across nurses, medical and allied health professions, coordinated responses to enhance team collaboration are warranted to retain early-career nurses in the profession.^84 85^

The role of managers is also of particular interest as they are best placed to listen to staff concerns and implement appropriate measures to prevent their departure. Staff are more likely to stay if they feel their recommendations are not ignored. It also increases the perception that their opinions positively contribute to the organisation.^82 86^ Managers and leaders also play a key role in the work culture, environment and relationships. The lack of perceived support from managers may, in contrast, lead to the departure of early-career nurses.^50^ Measures (and training) to ensure managers are accessible, compassionate and supportive are well within employers’ reach. They should continue to be prioritised to enhance the work environment and retain early-career nurses. What is less clear is how this guidance translates into what early-career nurses experience. This is especially critical in the post-COVID landscape, where nurses are grappling with heightened emotional and physical demands. The pandemic has highlighted even further the need for resilient workplace environments that not only retain but also protect and empower nurses at the start of their careers.^87 88^

Work pressure (ie, workload and intensity of the job)^41^ is clearly associated with poorer retention.^50 89^ Targeted interventions to reduce work pressure may well be the most challenging to address, reflecting structural factors and how workload, staffing and retention are intrinsically linked.^81 90^ Addressing nursing staff shortages is imperative to improving working conditions and, in turn, retention.^91 92^ While initiatives such as the N50k programme have increased the nursing workforce, longer-term success will be shown by building a sustainable nursing workforce, improving retention outcomes and safeguarding patient care.

One of our key findings was that early-career IENs also value good collaboration and teamwork, low work pressure, as well as opportunities for career development, consistent with findings elsewhere.^93^ Nurses recruited internationally tend to have several years of work experience before joining the UK, yet they start nursing in the UK on a Band 5-equivalent job—similar to what newly-graduated nurses would earn—which may not reflect their expertise, leading to frustration that their skills are not recognised and they may consider their work unchallenging.^94–96^ This may partially explain why career opportunities are highly valued among certain early-career IENs.^95 97^ These results relating to IENs should be interpreted cautiously. The qualitative work preceding the DCE did not include early-career IENs, so it is unclear to what extent the job attributes were relevant to them. For instance, attributes about their first experience of working in the UK and integration into the workplace may not have been captured.^96 97^ In addition, the majority of IENs were not ‘genuine’ early career nurses in the sense that they had more than 5 years of nursing experience on average. Nevertheless, the regression results between UK and IENs early-career NHS nurses were similar; the top four job attributes were the same across both groups. This suggests these results may—at least partially—reflect the expectations of ‘new’ nurses coming to work in the UK, and may warrant further exploratory work.

The study has several limitations. First, the DCE elicited stated preferences in hypothetical scenarios and therefore does not directly measure actual employment behaviour. Second, the choice tasks did not include an opt-out or status quo alternative. Although a no-choice option may increase behavioural realism, it can also increase task complexity and was not aligned with the study’s forced-choice comparison of job packages. Third, the DCE was limited to early-career nurses currently working in the NHS, which restricts generalisability to nurses employed in other sectors including the private and charity sectors. Nurses who have already left the NHS were also excluded. Further research should examine the preferences and experiences of these groups. Finally, the monetary estimates are marginal rates of substitution between salary and non-monetary attributes. They may overstate the salary changes required to alter actual employment decisions and should not be interpreted as literal out-of-pocket payments or as direct estimates of WTP compensation.^98^

## Conclusions

Retaining early-career nurses in the NHS is key to alleviating the nurse shortage and delivering safe and high-quality patient care. Using a DCE, this study identified all eight job attributes as significant determinants of job preferences among early-career NHS nurses. Notably, the results highlight the importance of good collaboration and teamwork, supportive and accessible managers and low work pressure as the top three most preferred job attributes. These findings were consistent across sensitivity analyses by region, healthcare sectors and country of first registration. Nurse leaders and decision-makers should therefore prioritise the development of supportive work environments characterised by strong teamwork, accessible leadership and manageable workloads to improve the retention of early-career nurses in the NHS.

## Supplementary material

10.1136/bmjopen-2026-118717 online supplemental file 1

10.1136/bmjopen-2026-118717 online supplemental file 2

10.1136/bmjopen-2026-118717 online supplemental file 3

10.1136/bmjopen-2026-118717 online supplemental file 4

## Data Availability

Data are available on reasonable request.

## References

[1] Kelly E, Stoye G, Warner M (2022). *Factors associated with staff retention in the nhs acute sector*.

[2] Nursing Times (2024). Rise in early career nurses leaving the profession.

[3] (2022). NHS Digital, HCHS Nurses and health visitors turnover by age and ethnicity.

[4] Medvec BR, Marriott DJ, Khadr L (2023). Patterns and Correlates of Nurse Departures From the Health Care Workforce: Results From a Statewide Survey. Med Care.

[5] Morgan B (2022). NHS staffing shortages: why do politicians struggle to give the NHS the staff it needs. The King’s Fund.

[6] Waitzman E (2022). Staff Shortages in the NHS and Social Care Sectors.

[7] (2023). NMC, Register leavers’ survey.

[8] Alarming increase in industrial action by nurses is a symptom of a global crisis in healthcare systems.

[9] Vázquez-Calatayud M, Eseverri-Azcoiti MC (2023). Retention of newly graduated registered nurses in the hospital setting: A systematic review. J Clin Nurs.

[10] Bae SH (2023). Comprehensive assessment of factors contributing to the actual turnover of newly licensed registered nurses working in acute care hospitals: a systematic review. BMC Nurs.

[11] Keith AC, Warshawsky N, Talbert S (2021). Factors That Influence Millennial Generation Nurses’ Intention to Stay. JONA.

[12] Ejebu O, Philippou J, Turnbull J (2024). Coming and going: A narrative review exploring the push-pull factors during nurses’ careers. Int J Nurs Stud.

[13] Ball J, Ejebu O Retention of NHS nurses: A scoping review.

[14] Ball J, Ejebu O, Saville C (2022). What keeps nurses in nursing? A scoping review into nurse retention. Nurs Times.

[15] Ejebu O (2025). Joining, staying or leaving: exploring the push-pull factors as nurses navigate through their careers. Evidence Brief.

[16] (2021). NHS England, Our NHS people promise.

[17] Conservative Party, Conservative Party Election Manifesto.

[18] Lancaster KJ (1966). A New Approach to Consumer Theory. Journal of Political Economy.

[19] McFadden D (1973). Conditional logit analysis of qualitative choice behavior.

[20] Hensher DA, Rose JM, Greene WH (2015). *Applied choice analysis*.

[21] Ejebu O, Turnbull J, Atherton I (2024). What might make nurses stay? A protocol for discrete choice experiments to understand NHS nurses’ preferences at early-career and late-career stages. BMJ Open.

[22] Li Y, Howell JR, Cimiotti JP (2023). Nurse practitioner job preference: A discrete choice experiment. Int J Nurs Stud.

[23] Cleland J, Porteous T, Ejebu O (2022). Won’t you stay just a little bit longer? A discrete choice experiment of UK doctors’ preferences for delaying retirement. Health Policy.

[24] Fields BE, Bell JF, Bigbee JL (2018). Registered nurses’ preferences for rural and urban jobs: A discrete choice experiment. Int J Nurs Stud.

[25] Scott A, Witt J, Duffield C (2015). What do nurses and midwives value about their jobs? Results from a discrete choice experiment. J Health Serv Res Policy.

[26] Mandeville KL, Lagarde M, Hanson K (2014). The use of discrete choice experiments to inform health workforce policy: a systematic review. BMC Health Serv Res.

[27] de Bekker-Grob EW, Ryan M, Gerard K (2012). Discrete choice experiments in health economics: a review of the literature. Health Econ.

[28] Ejebu O, Ball J, Jaffry S Late-career nurses matter: a discrete choice experiment to elicit preferences to delay retirement, International Journal of Nursing Studies, 2026, Accepted for Publication.

[29] Smitz M-F, Witter S, Lemiere C (2016). Understanding Health Workers’ Job Preferences to Improve Rural Retention in Timor-Leste: Findings from a Discrete Choice Experiment. PLoS One.

[30] Song K, Scott A, Sivey P (2015). Improving Chinese primary care providers’ recruitment and retention: a discrete choice experiment. Health Policy Plan.

[31] Kunaviktikul W, Chitpakdee B, Srisuphan W (2015). Preferred choice of work setting among nurses in Thailand: A discrete choice experiment. Nurs Health Sci.

[32] Robyn PJ, Shroff Z, Zang OR (2015). Addressing health workforce distribution concerns: a discrete choice experiment to develop rural retention strategies in Cameroon. Int J Health Policy Manag.

[33] Rao KD, Ryan M, Shroff Z (2013). Rural clinician scarcity and job preferences of doctors and nurses in India: a discrete choice experiment. PLoS One.

[34] Rockers PC, Jaskiewicz W, Kruk ME (2013). Differences in preferences for rural job postings between nursing students and practicing nurses: evidence from a discrete choice experiment in Lao People’s Democratic Republic. Hum Resour Health.

[35] Doiron D, Hall J, Kenny P (2014). Job preferences of students and new graduates in nursing. Appl Econ.

[36] Lagarde M, Blaauw D (2009). A review of the application and contribution of discrete choice experiments to inform human resources policy interventions. Hum Resour Health.

[37] Ryan M, Gerard K, Amaya-Amaya M (2008). Using discrete choice experiments to value health and health care.

[38] Lancsar E, Louviere J (2008). Conducting discrete choice experiments to inform healthcare decision making: a user’s guide. Pharmacoeconomics.

[39] Ball J, Turnbull J, Ejebu O (2026). Dataset supporting an article: Nurses’ perspectives regarding their employers’ efforts to recruit and retain them: a cross-sectional survey in England.

[40] Malterud K, Siersma VD, Guassora AD (2016). Sample Size in Qualitative Interview Studies: Guided by Information Power. Qual Health Res.

[41] Ejebu O, Ellis J, Sun L (2025). Values and Workplace Expectations to Facilitate Retention: Perspectives From Nurses at Two Ends of the Career Spectrum. J Nurs Manag.

[42] Gularte-Rinaldo J, Baumgardner R, Tilton T (2023). Mentorship ReSPeCT Study: A Nurse Mentorship Program’s Impact on Transition to Practice and Decision to Remain in Nursing for Newly Graduated Nurses. Nurse Lead.

[43] Brook J, Aitken L, Webb R (2019). Characteristics of successful interventions to reduce turnover and increase retention of early career nurses: A systematic review. Int J Nurs Stud.

[44] HEE (2015). RePAIR - Executive Summary.

[45] Kaldal MH, Voldbjerg SL, Grønkjaer M (2024). Newly graduated nurses’ commitment to the nursing profession and their workplace during their first year of employment: A focused ethnography. J Adv Nurs.

[46] Yarbrough S, Martin P, Alfred D (2017). Professional values, job satisfaction, career development, and intent to stay. Nurs Ethics.

[47] Jones SJ (2017). Establishing a Nurse Mentor Program to Improve Nurse Satisfaction and Intent to Stay. J Nurses Prof Dev.

[48] Sandler M (2018). Why are new graduate nurses leaving the profession in their first year of practice and how does this impact on ED nurse staffing? A rapid review of current literature and recommended reading. *CJEN*.

[49] Jiang L, Xu XV, Jacobs S (2023). From Incivility to Turnover Intentions among Nurses: A Multifoci and Self-Determination Perspective. J Nurs Manag.

[50] Çamveren H, Arslan Yürümezoğlu H, Kocaman G (2020). Why do young nurses leave their organization? A qualitative descriptive study. Int Nurs Rev.

[51] Lavoie-Tremblay M, Fernet C, Lavigne GL (2016). Transformational and abusive leadership practices: impacts on novice nurses, quality of care and intention to leave. J Adv Nurs.

[52] Pressley C, Garside J (2023). Safeguarding the retention of nurses: A systematic review on determinants of nurse’s intentions to stay. Nurs Open.

[53] Richard E, Kim SBC (2024). Career decisions and aspirations of early-career nurses: Insights from a qualitative interpretative description study. J Adv Nurs.

[54] Wu T-Y, Fox DP, Stokes C (2012). Work-related stress and intention to quit in newly graduated nurses. Nurse Educ Today.

[55] Kim Y, Kim H-Y (2021). Retention Rates and the Associated Risk Factors of Turnover among Newly Hired Nurses at South Korean Hospitals: A Retrospective Cohort Study. IJERPH.

[56] Dwyer PA, Hunter Revell SM, Sethares KA (2019). The influence of psychological capital, authentic leadership in preceptors, and structural empowerment on new graduate nurse burnout and turnover intent. Appl Nurs Res.

[57] Favaro A, Wong C, Oudshoorn A (2021). Relationships among sex, empowerment, workplace bullying and job turnover intention of new graduate nurses. J Clin Nurs.

[58] Propper C, Stockton I, Stoye G Cost of Living and the Impact on Nursing Labour Outcomes in NHS Acute Trusts.

[59] (2020). Ngene. http://www.choice-metrics.com/contact.html.

[60] Bliemer MCJ, Collins AT (2016). On determining priors for the generation of efficient stated choice experimental designs. *Journal of Choice Modelling*.

[61] Mühlbacher A, Johnson FR (2016). Choice Experiments to Quantify Preferences for Health and Healthcare: State of the Practice. Appl Health Econ Health Policy.

[62] Huber J, Zwerina K (1996). The Importance of Utility Balance in Efficient Choice Designs. *Journal of Marketing Research*.

[63] Louviere JJ, Hensher DA, Swait JD (2000). Stated Choice Methods: Analysis and Applications.

[64] de Bekker-Grob EW, Donkers B, Jonker MF (2015). Sample Size Requirements for Discrete-Choice Experiments in Healthcare: a Practical Guide. *Patient*.

[65] Weber S (2021). A Step-by-Step Procedure to Implement Discrete Choice Experiments in Qualtrics. Soc Sci Comput Rev.

[66] (2020). Qualtrics.

[67] Turnbull J (2025). Evaluation of the N50k programme to expand the NHS nursing workforce.

[68] Turnbull J (2026). Expanding the NHS Nursing Workforce: retention and recruitment.

[69] Ryan M (2012). How to Conduct a Discrete Choice Experiment for Health Workforce Recruitment and Retention in Remote and Rural Areas: A User Guide with Case Studies.

[70] Illman J (2023). Almost all promised ‘50,000 more nurses’ are international recruits. HSJ.

[71] Midwifery Council (2023). Record 808,488 nurses, midwives and nursing associates in the UK.

[72] (2019). The Economic and Social Research Council The use of incentives to recruit and retain hard-to-get populations in longitudinal studies.

[73] Bliemer MC, Rose JM (2024). Designing and Conducting Stated Choice Experiments, in Handbook of Choice Modelling.

[74] Moradi K, Khosravi K, Jalali A (2025). From classroom to workforce: a discrete choice analysis of Iranian nursing students’ job preferences. BMC Med Educ.

[75] (2025).

[76] (2025). NMC. England permanent register data table.

[77] Shembavnekar N, Buchan J (2022). How reliant is the NHS in England on international nurse recruitment. The Health Foundation.

[78] NHS Employers (2021). NHS Terms and Conditions (AfC) pay scales - Annual.

[79] Heyes A (2005). The economics of vocation or “why is a badly paid nurse a good nurse”?. J Health Econ.

[80] Williamson GR, Health V, Proctor-Childs T (2013). Vocation, friendship and resilience: a study exploring nursing student and staff views on retention and attrition. Open Nurs J.

[81] Marufu TC, Collins A, Vargas L (2021). Factors influencing retention among hospital nurses: systematic review. Br J Nurs.

[82] Africa L, Trepanier S (2021). The Role of the Nurse Leader in Reversing the New Graduate Nurse Intent to Leave. Nurse Lead.

[83] Chan ZCY, Tam WS, Lung MKY (2013). A systematic literature review of nurse shortage and the intention to leave. J Nurs Manag.

[84] Andrews MC, Woolum A, Mesmer-Magnus J (2024). Reducing turnover intentions among first-year nurses: The importance of work centrality and coworker support. Health Serv Manage Res.

[85] Brewer CS, Chao Y-Y, Colder CR (2015). A structural equation model of turnover for a longitudinal survey among early career registered nurses. Int J Nurs Stud.

[86] Al-Amer R, Darwish M, Malak M (2022). Nurses experience of caring for patients with COVID-19: A phenomenological study. Front Psychiatry.

[87] Abuzied Y, Al-Amer R, Abuzaid M (2022). The Magnet Recognition Program and Quality Improvement in Nursing. *Glob J Qual Saf Healthc*.

[88] Abuzied Y, Al-Amer R, Somduth S (2021). Psychological Responses Among Healthcare Workers Providing Care for Patients with COVID-19: A Web-Based Cross-Sectional Survey in Riyadh, Saudi Arabia. *Glob J Qual Saf Healthc*.

[89] An M, Heo S, Hwang YY (2022). Factors Affecting Turnover Intention among New Graduate Nurses: Focusing on Job Stress and Sleep Disturbance. Health Care (Don Mills).

[90] Hart SM, Warren AM (2015). Understanding nurses’ work: Exploring the links between changing work, labour relations, workload, stress, retention and recruitment. *Economic and Industrial Democracy*.

[91] Griffiths P, Recio-Saucedo A, Dall’Ora C (2018). The association between nurse staffing and omissions in nursing care: A systematic review. J Adv Nurs.

[92] Duffield C, Diers D, O’Brien-Pallas L (2011). Nursing staffing, nursing workload, the work environment and patient outcomes. Appl Nurs Res.

[93] Omiyi D, Wilkinson E, Snaith B (2025). Exploring the Motivations, Challenges, and Integration of Internationally Educated Healthcare Workers in the UK: A Scoping Review. *Policy, Politics, & Nursing Practice*.

[94] Bond S, Merriman C, Walthall H (2020). The experiences of international nurses and midwives transitioning to work in the UK: A qualitative synthesis of the literature from 2010 to 2019. Int J Nurs Stud.

[95] Garside JR (2023). International nurses and their initial integration into NHS England’s healthcare workforce: a population analysis. Health and Wellbeing Academy.

[96] Pressley C, Newton D, Garside J (2022). Global migration and factors that support acculturation and retention of international nurses: A systematic review. Int J Nurs Stud Adv.

[97] Pressley C, Newton D, Sanderson L (2025). International Nurse Migration Experience of the First Two Years: A Mixed Methods Study. J Adv Nurs.

[98] Allenby GM, Brazell JD, Howell JR (2014). Economic valuation of product features. *Quant Mark Econ*.

