# Supplementary material for: Making nursing a better place: a discrete choice experiment to elicit the job preferences of early-career nurses in England
Source: BMJ Open. 2026 Sep 25;16(9):e118717. doi: 10.1136/bmjopen-2026-118717 (PMC13629952; doi:10.1136/bmjopen-2026-118717)
Supplement: Supplementary file 1 [file bmjopen-2026-118717.reviewer_comments.pdf]

PEER REVIEW HISTORY

BMJ Open publishes all reviews undertaken for accepted manuscripts. Reviewers are asked to complete a checklist review form and are provided with free text boxes to elaborate on their assessment. These free text comments are reproduced below.

ARTICLE DETAILS

Title (Provisional)

Making Nursing a Better Place: A Discrete Choice Experiment to Elicit the Job Preferences of Early-Career Nurses in England

Authors

Ejebu, Ourega-Zoé; Ball, Jane; Genie, Mesfin G; Philippou, Julia; Turnbull, Joanne

---

VERSION 1 - REVIEW

**Reviewer**	**1**
**Name**	**Sayles, Harlan**
**Affiliation**	**University of Nebraska Medical Center, Biostatistics**
**Date**	**22-Apr-2026**
**COI**	**None**

This is a very well-written manuscript detailing a well-planned and executed study of job characteristic preferences among early-career nurses in the UK. As such, I only have a few minor comments below.

Page 7, lines 26-30: A sample of 125 would allow you to estimate your regression coefficients with what level of precision? This is not critically important as your sample size is much larger than 125, but when we do power analysis, we usually report a level of precision.

Page 8, line 38 contains a random(?) question mark.

I would add as a limitation in the discussion relating to generalizability that you only studied working nurses and the main part of the study (the DCE) was limited to NHS nurses. I think an obvious follow-up study, which may be somewhat difficult to find a target population for, would be surveying nurses who did not progress past the early part of their career. It would also be of interest if you could track your respondents and follow-up with them in 5 years to see how their responses to the study relate to whether they are still working as nurses in the NHS. You may be able to accomplish both goals if you can interview some of the nurses who have left to understand why they left.

**Reviewer**	**2**
**Name**	**Abuzied, Yacoub**
**Affiliation**	**King Fahad Medical City, Nursing**
**Date**	**29-May-2026**
**COI**	**None**

This manuscript addresses a highly relevant and timely issue in nursing workforce management. Understanding the job preferences of early-career nurses is essential for improving recruitment, retention, and job satisfaction, particularly in the context of ongoing nursing shortages. The study provides valuable insights that may inform workforce policies and organizational strategies. Overall, the manuscript is well written and has the potential to make a meaningful contribution to the nursing and healthcare management literature. Please find below my comments and suggestions to further strengthen the paper.

- Since the study incorporates both qualitative and quantitative components, the authors may consider indicating the mixed-methods design in the title to better reflect the study methodology. "Making Nursing a Better Place: A Mixed-Methods Discrete Choice Experiment to Elicit the Job Preferences of Early-Career Nurses".

- Please state the study design, the number of participants in each phase, and the main qualitative themes identified in the Abstract.

- As part of the limitations of the study is "Nurses who have left the NHS were not included in the study", it gives a power to initiate further research on understanding the issue. If this sentence can be extended to give a recommendation will be sound good.

- P5, Line 20 and from P7, line 10, , Please clearly state the study design and explain how the qualitative findings were used to develop the DCE survey and choice sets generated using Ngene software.

- P5, line 29, The authors are encouraged to provide further details regarding the qualitative sample size. Specifically, how were the 11 participants recruited and selected, and what rationale was used to determine that this number was sufficient?

In addition, please clarify whether data saturation was assessed and achieved, and describe the process used to determine saturation. This information would strengthen the methodological rigor and trustworthiness of the qualitative findings.

- P10, Lines 36-37, the authors mentioned "36.5% of the respondents were White, 35.9% were Asian/British, and 21.2% were Black/British". > As all participants were recruited from the United Kingdom, the rationale for reporting ethnicity as White, Black, and Asian should be clarified. If ethnicity is relevant to the study objectives or analysis, please explain its

inclusion and discuss its potential influence on job preferences. Otherwise, consider whether these demographic categories are necessary to report in detail.

- The qualitative phase is not sufficiently described. Please provide the main themes and subthemes that emerged from the interviews and explain how they informed the selection of the DCE attributes and levels. As the qualitative findings formed the basis of the DCE design, a brief presentation of the key themes and evidence of data saturation would strengthen the transparency and methodological rigor of the study.

- P16, Lines 48-9, the authors mentioned "The role of managers is also of particular interest as they are best placed to listen to staff concerns and implement appropriate measures to prevent their departure." You might cite it with the following reference:

Abuzied Y, Al-Amer R, Abuzaid M, Somduth S. The magnet recognition program and quality improvement in nursing. Global journal on quality and safety in healthcare. 2022 Nov 1;5(4):106-8.

- P17, Lines 2-7, the authors mentioned "This is especially critical in the postCOVID landscape, where nurses are grappling with heightened emotional and physical demands. The pandemic has highlighted even further the need for resilient workplace environments that not only retain but also protect and empower nurses at the start of their careers." I suggest adding the following references concerning this issue as follows:

Al-Amer R, Darwish M, Malak M, Ali AM, Al Weldat K, Alkhamees A, Alshammari KS, Abuzied Y, Randall S. Nurses experience of caring for patients with COVID-19: A phenomenological study. Frontiers in Psychiatry. 2022 Jul 19;13:922410.

Abuzied Y, Al-Amer R, Somduth S, Silva G, Muthuraj A, AlEnizi S, AlGhamdi K. Psychological responses among healthcare workers providing care for patients with COVID-19: a web-based cross-sectional survey in Riyadh, Saudi Arabia. Global Journal on Quality and Safety in Healthcare. 2021 Nov 1;4(4):131-4.

- Again, this should be acknowledged and discussed as part of the study limitations, as mentioned earlier "Nurses who have left the NHS were not included in the study", It may serve as a basis for further research on this topic.

- Please expand the conclusion to better reflect and summarize the key findings of the study.

- Figure 2, Please improve the image resolution and clarity.

I hope these comments and suggestions will help the authors further strengthen the manuscript.

VERSION 1 - AUTHOR RESPONSE

We are grateful to both reviewers for their comments, which greatly contributed to enhancing our paper. We have addressed them and highlighted the changes in yellow in the manuscript.

Reviewers’ comments	Authors’ responses
**Reviewer 1**	
Page 7, lines 26-30: A sample of 125 would allow you to estimate your regression coefficients with what level of precision? This is not critically important, as your sample size is much larger than 125, but when we do power analysis, we usually report a level of precision.	Thank you for your comment. The Louviere formula is a heuristic sample-size guideline rather than a power calculation. No explicit level of precision can be inferred from the Louviere formula. Therefore, the study's precision is assessed from the estimated standard errors and confidence intervals obtained after the model estimation. We’ve clarified this on **page 6**, as follows:<br>As the Louviere formula is a heuristic sample-size guideline rather than a power calculation, an explicit level of precision cannot be inferred. Therefore, the study's precision is obtained from the estimated standard errors and confidence intervals of the regression models (de Bekker-Grob et al., 2015).
Page 8, line 38 contains a random(?) question mark.	Thank you for your comments. We’ve now removed the interrogation mark on **page 7**.
I would add as a limitation in the discussion relating to generalizability that you only studied working nurses and the main part of the study (the DCE) was limited to NHS nurses. I think an obvious follow-up study, which may be somewhat difficult to find a target population for, would be surveying nurses who did not progress past the early part of their career. It would also be of interest if you could track your respondents and follow-up with them in 5 years to see how their responses to the study relate to whether they are still working as nurses in the NHS. You may be able to accomplish both goals if you can interview some of the nurses who have left to understand why they left.	Thank you for your comments. We’ve added the comment about nurses from other sectors not being included, and the implications for results interpretation on **page 18**, as follows:<br>The DCE was limited to early-career nurses working in the NHS, limiting the generalisability to other sectors, including the private and charity sectors.<br><br>We absolutely agree that a follow-up survey would be very insightful to understand the reasons for leaving. However, the survey was anonymous, as we unfortunately did not collect information to follow up with respondents. But we added this as a recommendation for further research in the limitation section on **page 18**, as follows:<br>Nurses who have left the NHS were not included in the study, so the results may not be generalisable to this group. Further research could focus on such nurses to understand the reasons for their departure and potentially prevent further ones.
**Reviewer 2**	**Authors’ responses**
- Since the study incorporates both qualitative and quantitative components, the authors may consider indicating the mixed-methods design in the title to better reflect	Thank you for your comments. We’ve now amended the **title** to reflect both qualitative and quantitative approaches in our DCE study.

the study methodology. "Making Nursing a Better Place: A Mixed-Methods Discrete Choice Experiment to Elicit the Job Preferences of Early-Career Nurses".	
- Please state the study design, the number of participants in each phase, and the main qualitative themes identified in the Abstract.	Thank you for your comment. We've now added the number of participants and clarified the study design in the **abstract**, as follows:<br>11 early-career nurses participated in the focus groups.<br><br>The design was clarified in the **abstract** as follows:<br>A cross-sectional Discrete Choice Experiment (DCE) administered through online surveys distributed by the Nursing and Midwifery Council.
As part of the limitations of the study is "Nurses who have left the NHS were not included in the study", it gives a power to initiate further research on understanding the issue. If this sentence can be extended to give a recommendation will be sound good.	Thank you for your comment. BMJ Open's rules about limitation statements do not permit adding a recommendation, so it was added in the limitation section instead on **page 18**.<br>Nurses who have left the NHS were not included in the study, so the results may not be generalisable to this group. Further research could focus on such nurses to understand the reasons for their departure and potentially prevent further ones.
- P5, Line 20 and from P7, line 10, Please clearly state the study design and explain how the qualitative findings were used to develop the DCE survey and choice sets generated using Ngene software.	Thank you for your comment. We've added the study design on **page** 7.<br><br>We've revisited this section and are confident that the 8 DCE attributes are explicitly described on **pages 4-5** with rationales and references for inclusion.
- P5, line 29, The authors are encouraged to provide further details regarding the qualitative sample size. Specifically, how were the 11 participants recruited and selected, and what rationale was used to determine that this number was sufficient? In addition, please clarify whether data saturation was assessed and achieved, and describe the process used to determine saturation. This information would strengthen the methodological rigor and trustworthiness of the qualitative findings.	Thank you for your comment. We've provided more information on the qualitative design of the DCE, and made a reference to the qualitative study that was purposely designed for the DCE .<br><br>The section reads as follows on **page 4**:<br>Online focus groups and interviews were conducted with 11 early-career nurses working in NHS England. Participants were recruited via several channels, including social media and an earlier survey conducted in 2023, where respondents could agree to be contacted for future research (Ball et al., 2026). We purposively sampled nurses to represent different demographic

	characteristics and job roles and capture a diversity of experiences and views across participants. Recruitment was conducted until reaching data saturation, that is, until we were confident that we had collected sufficient data to yield enough information power to elucidate the aims of the study (Malterud et al., 2016). More details on the qualitative study findings can be found elsewhere (Ejebu et al., 2025).
- P10, Lines 36-37, the authors mentioned "36.5% of the respondents were White, 35.9% were Asian/British, and 21.2% were Black/British". As all participants were recruited from the United Kingdom, the rationale for reporting ethnicity as White, Black, and Asian should be clarified. If ethnicity is relevant to the study objectives or analysis, please explain its inclusion and discuss its potential influence on job preferences. Otherwise, consider whether these demographic categories are necessary to report in detail.	Thank you for your comment. The inclusion of demographic statistics by ethnicity was included to capture the fact that the majority of early-career nurses who registered between 2019 and 2024 were recruited internationally. Indeed, this DCE study was part of a larger project that evaluated the Government's initiative to increase the NHS workforce by 50,000 by 2019. 84% (43,000) of the 50,000 nurses were recruited internationally. We believe it was important to verify and confirm this trend in our data. We clarified that in the following sections, as follows:<br>**Page 7**<br>Since the study was part of a larger project (i.e. N50K Nurses Programme) the survey was sent to all nurses who first registered with the NMC between 2019 and 2024 and were working in England at the time of the study (N=143,133). This also includes internationally-educated nurses (IENs) who were recruited in large numbers as part of the N50k programme (Illman, 2023, Nursing & Midwifery Council, 2023).<br><br>**Page 9**<br>Half (50.4%) of the respondents first registered in the UK, 31.9% first registered in Asia (predominantly Philippines and India – 93.7% in total), and 14.4% in Africa and the Caribbean Islands (predominantly Ghana and Nigeria – 65.3% in total), reflecting the patterns of the N50k international recruitment initiative (Illman, 2023).
- The qualitative phase is not sufficiently described. Please provide the main themes and subthemes that emerged from the interviews and explain how they informed the selection of the DCE attributes and levels. As the qualitative findings formed the basis of	Thank you for your comments. We believe we've now addressed the description of the qualitative study in the previous comment.<br><br>We revisited the section on qualitative findings and believe the 8 DCE attributes

the DCE design, a brief presentation of the key themes and evidence of data saturation would strengthen the transparency and methodological rigor of the study.	were already explicitly described on **pages 4-5,** with rationales for inclusion and relevant references.
- P16, Lines 48-9, the authors mentioned "The role of managers is also of particular interest as they are best placed to listen to staff concerns and implement appropriate measures to prevent their departure." You might cite it with the following reference: Abuzied Y, Al-Amer R, Abuzaid M, Somduth S. The magnet recognition program and quality improvement in nursing. Global journal on quality and safety in healthcare. 2022 Nov 1;5(4):106-8.	Thank you for your suggestion. We've added the reference on **page 16.**
- P17, Lines 2-7, the authors mentioned "This is especially critical in the post-COVID landscape, where nurses are grappling with heightened emotional and physical demands. The pandemic has highlighted even further the need for resilient workplace environments that not only retain but also protect and empower nurses at the start of their careers." I suggest adding the following references concerning this issue as follows: Al-Amer R, Darwish M, Malak M, Ali AM, Al Weldat K, Alkhamees A, Alshammari KS, Abuzied Y, Randall S. Nurses experience of caring for patients with COVID-19: A phenomenological study. Frontiers in Psychiatry. 2022 Jul 19;13:922410.<br><br>Abuzied Y, Al-Amer R, Somduth S, Silva G, Muthuraj A, AlEnizi S, AlGhamdi K. Psychological responses among healthcare workers providing care for patients with COVID-19: a web-based cross-sectional survey in Riyadh, Saudi Arabia. Global Journal on Quality and Safety in Healthcare. 2021 Nov 1;4(4):131-4.	Thank you for your suggestion. We've added both references on **page 17.**
- Again, this should be acknowledged and discussed as part of the study limitations, as mentioned earlier "Nurses who have left the NHS were not included in the study", It may serve as a basis for further research on this topic.	Thank you for your comment. BMJ Open's rules about limitation statements do not permit adding a recommendation, so it was added in the limitation section instead on **page 17**:<br>Nurses who have left the NHS were not included in the study, so the results may not be generalisable to this group. Further research could focus on such nurses to understand the reasons for their departure and potentially prevent further ones.

- Please expand the conclusion to better reflect and summarize the key findings of the study.	Thank you for your comments. We've amended the conclusion to better reflect the findings of the study on **page 18**:<br>Retaining early-career nurses in the NHS is key to alleviating the nurse shortage and delivering safe and high-quality patient care. Using a Discrete Choice Experiment, this study identified all eight job attributes as significant determinants of job preferences among early-career NHS nurses. Notably, the results highlight the importance of good collaboration and teamwork, supportive and accessible managers and low work pressure as the top three most preferred job attributes. These findings were consistent across sensitivity analyses by region, healthcare sectors and country of first registration. Nurse leaders and decision-makers should therefore prioritise the development of supportive work environments characterised by strong teamwork, accessible leadership and manageable workloads to improve the retention of early-career nurses in the NHS.
Figure 2, Please improve the image resolution and clarity.	Thank you for your comment. We've provided an enhanced version of Figure 2.
