# Supplementary material for: Making nursing a better place: a discrete choice experiment to elicit the job preferences of early-career nurses in England
Source: BMJ Open. 2026 Sep 25;16(9):e118717. doi: 10.1136/bmjopen-2026-118717 (PMC13629952; doi:10.1136/bmjopen-2026-118717)
Supplement: Supplementary file 2 [file bmjopen-16-9-s001.docx]

**Appendix 1**

**Table 7: Correlation table and significance level of the DCE attributes**

|  | A | B | C | D | E | F | G | H |
| --- | --- | --- | --- | --- | --- | --- | --- | --- |
| Career development (A) | 1 |  |  |  |  |  |  |  |
|  |  |  |  |  |  |  |  |  |
| Organisational support (B) | 0.145 | 1 |  |  |  |  |  |  |
|  | 0.000 |  |  |  |  |  |  |  |
| Collaboration & teamwork (C) | 0.143 | 0.287 | 1 |  |  |  |  |  |
|  | 0.000 | 0.000 |  |  |  |  |  |  |
| Managers (D) | 0.141 | 0.145 | -0.143 | 1 |  |  |  |  |
|  | 0.000 | 0.000 | 0.000 |  |  |  |  |  |
| Flexible work patterns (E) | 0.145 | 0.145 | 0.143 | 0.141 | 1 |  |  |  |
|  | 0.000 | 0.000 | 0.000 | 0.000 |  |  |  |  |
| Work pressure (F) | 0.145 | 0.141 | -0.287 | 0.145 | 0.141 | 1 |  |  |
|  | 0.000 | 0.000 | 0.000 | 0.000 | 0.000 |  |  |  |
| Recognition (G) | 0.141 | 0.141 | 0.140 | 0.145 | 0.145 | 0.145 | 1 |  |
|  | 0.000 | 0.000 | 0.000 | 0.000 | 0.000 | 0.000 |  |  |
| Salary (H) | 0.170 | 0.335 | 0.101 | 0.335 | 0.168 | 0.170 | 0.168 | 1 |
|  | 0.000 | 0.000 | 0.000 | 0.000 | 0.000 | 0.000 | 0.000 |  |

nurses_Supplemental
