# Supplementary material for: Making nursing a better place: a discrete choice experiment to elicit the job preferences of early-career nurses in England
Source: BMJ Open. 2026 Sep 25;16(9):e118717. doi: 10.1136/bmjopen-2026-118717 (PMC13629952; doi:10.1136/bmjopen-2026-118717)
Supplement: Supplementary file 3 [file bmjopen-16-9-s002.docx]

**Appendix 2**

**CHOICE 1**

If you were offered these two nursing jobs, which job would you choose?

| **Job characteristics** | **Job A** | **Job B** |
| --- | --- | --- |
| Career development | Good opportunities for career development | Limited opportunities for career development |
| Organisational support | Good organisational support, well structured | Lack of organisational support, poorly structured |
| Teamwork | Poor collaboration and teamwork | Poor collaboration and teamwork |
| Management | Supportive and accessible | Unsupportive and inaccessible |
| Personalised working | Lack of flexible working patterns | Good flexible working patterns |
| Work pressure | Low work pressure | High work pressure |
| Recognition | Low recognition, skills not valued | Good recognition, skills are valued |
| Salary | 10% increase in income | 10% increase in income |

Preferred job: ❑ Job A	❑ Job B

**Figure 1: Example of the DCE job choice to explore the job preferences among early-career NHS nurses**
