# Supplementary material for: Making nursing a better place: a discrete choice experiment to elicit the job preferences of early-career nurses in England
Source: BMJ Open. 2026 Sep 25;16(9):e118717. doi: 10.1136/bmjopen-2026-118717 (PMC13629952; doi:10.1136/bmjopen-2026-118717)
Supplement: Supplementary file 4 [file bmjopen-16-9-s003.docx]

**Appendix 3**

**Table 8:** **Results of Mixed Logit regressions to explore the job preferences of early-career NHS nurses: by region**

|  | **East of England** | | **London** | | **Midland** | | **North East & Yorkshire** | | **North West** | | **South East** | | **South West** | |
| --- | --- | --- | --- | --- | --- | --- | --- | --- | --- | --- | --- | --- | --- | --- |
|  | Mean | SD | Mean | SD | Mean | SD | Mean | SD | Mean | SD | Mean | SD | Mean | SD |
| **Good collaboration and teamwork** | 0.990*** | -0.311 | 1.093*** | 0.000 | 1.149*** | 0.052 | 1.314*** | -0.002 | 1.267*** | 0.007 | 1.312*** | 0.388 | 1.328*** | 0.400 |
| *(Ref: Poor collaboration and teamwork)* | [0.105] | [0.242] | [0.083] | [0.379] | [0.079] | [0.391] | [0.100] | [0.444] | [0.110] | [0.210] | [0.103] | [0.282] | [0.108] | [0.290] |
| **Supportive and accessible managers** | 0.750*** | -0.013 | 0.751*** | -0.011 | 0.811*** | 0.164 | 0.863*** | -0.001 | 0.896*** | -0.131 | 0.866*** | 0.232 | 0.891*** | -0.015 |
| *(Ref: Unsupportive and inaccessible managers)* | [0.065] | [0.200] | [0.050] | [0.199] | [0.049] | [0.190] | [0.060] | [0.319] | [0.069] | [0.271] | [0.062] | [0.176] | [0.064] | [0.207] |
| **Low work pressure** | 0.627*** | 0.442*** | 0.684*** | 0.434*** | 0.758*** | 0.585*** | 0.804*** | 0.468*** | 0.858*** | 0.538*** | 0.851*** | 0.550*** | 0.835*** | 0.530*** |
| *(Ref: High work pressure)* | [0.074] | [0.115] | [0.060] | [0.086] | [0.057] | [0.074] | [0.069] | [0.094] | [0.076] | [0.100] | [0.073] | [0.108] | [0.075] | [0.109] |
| **Good opportunities for career development** | 0.761*** | 0.545*** | 0.708*** | 0.297*** | 0.688*** | 0.509*** | 0.667*** | 0.411*** | 0.610*** | 0.469*** | 0.807*** | 0.540*** | 0.757*** | 0.529*** |
| *(Ref: Limited opportunities for career development)* | [0.061] | [0.102] | [0.045] | [0.115] | [0.044] | [0.077] | [0.051] | [0.102] | [0.054] | [0.104] | [0.057] | [0.093] | [0.057] | [0.097] |
| **Flexible working patterns** | 0.514*** | 0.312** | 0.477*** | -0.004 | 0.574*** | -0.024 | 0.583*** | 0.334*** | 0.627*** | 0.267* | 0.686*** | 0.459*** | 0.698*** | 0.155 |
| *(Ref: Lack of flexible working patterns)* | [0.055] | [0.144] | [0.041] | [0.207] | [0.039] | [0.449] | [0.048] | [0.116] | [0.053] | [0.145] | [0.055] | [0.104] | [0.055] | [0.254] |
| **Good recognition, skills are valued** | 0.544*** | 0.054 | 0.491*** | -0.002 | 0.523*** | 0.269** | 0.626*** | -0.132 | 0.600*** | 0.158 | 0.630*** | -0.269 | 0.654*** | 0.249 |
| *(Ref: Low recognition, skills are not valued)* | [0.053] | [0.308] | [0.041] | [0.197] | [0.040] | [0.124] | [0.048] | [0.261] | [0.050] | [0.231] | [0.051] | [0.212] | [0.054] | [0.252] |
| **Good organisational support, well structured** | 0.549*** | 0.462*** | 0.428*** | 0.182 | 0.452*** | 0.451*** | 0.378*** | 0.383*** | 0.468*** | 0.369*** | 0.539*** | 0.421*** | 0.491*** | 0.298** |
| *(Ref: Lack of organisational support, poorly structured)* | [0.058] | [0.108] | [0.044] | [0.181] | [0.043] | [0.083] | [0.049] | [0.107] | [0.052] | [0.117] | [0.052] | [0.109] | [0.053] | [0.142] |
| **Salary** | 0.036*** |  | 0.041*** |  | 0.044*** |  | 0.042*** |  | 0.038*** |  | 0.049*** |  | 0.047*** |  |
|  | [0.003] |  | [0.002] |  | [0.002] |  | [0.002] |  | [0.003] |  | [0.003] |  | [0.003] |  |
| **Constant (Job Choice A)** | 0.202*** | -0.047 | 0.209*** | 0.001 | 0.213*** | -0.002 | 0.166*** | 0.000 | 0.148*** | 0.149 | 0.191*** | 0.008 | 0.208*** | 0.017 |
|  | [0.047] | [0.505] | [0.040] | [0.122] | [0.036] | [0.108] | [0.043] | [0.174] | [0.042] | [0.268] | [0.043] | [0.135] | [0.046] | [0.240] |
| Observations | 7,308 |  | 9,528 |  | 12,780 |  | 9,012 |  | 9,792 |  | 10,404 |  | 8,760 |  |
| Number of respondents | 609 |  | 794 |  | 1,065 |  | 751 |  | 816 |  | 867 |  | 730 |  |
| ***p<0.01, **p<0.05  Standard errors in squared brackets | | | | | | | | | | | | | | |

|  | East of England | London | Midland | North East & Yorkshire | North West | South East | South West |
| --- | --- | --- | --- | --- | --- | --- | --- |
| Good collaboration and teamwork | -27.5% | -26.7% | -26.1% | -31.3% | -33.3% | -26.8% | -28.3% |
| Supportive and accessible managers | -20.8% | -18.3% | -18.4% | -20.5% | -23.6% | -17.7% | -19.0% |
| Low work pressure | -17.4% | -16.7% | -17.2% | -19.1% | -22.6% | -17.4% | -17.8% |
| Good opportunities for career development | -21.1% | -17.3% | -15.6% | -15.9% | -16.1% | -16.5% | -16.1% |
| Flexible working patterns | -14.3% | -11.6% | -13.0% | -13.9% | -16.5% | -14.0% | -14.9% |
| Good recognition, skills are valued | -15.1% | -12.0% | -11.9% | -14.9% | -15.8% | -12.9% | -13.9% |
| Good organisational support, well structured | -15.3% | -10.4% | -10.3% | -9.0% | -12.3% | -11.0% | -10.4% |

**Table 8: WTP (%) of early-career NHS nurses by region**
