# Supplementary material for: Making nursing a better place: a discrete choice experiment to elicit the job preferences of early-career nurses in England
Source: BMJ Open. 2026 Sep 25;16(9):e118717. doi: 10.1136/bmjopen-2026-118717 (PMC13629952; doi:10.1136/bmjopen-2026-118717)
Supplement: Supplementary file 5 [file bmjopen-16-9-s004.docx]

**Appendix 4**

**Table 9: Results of Mixed Logit regressions to explore the job preferences of early-career NHS nurses: healthcare sectors, WTP and equivalent in annual average income (£)**

| Job characteristics | **Primary Care sector** | | **Secondary Care sector** | |
| --- | --- | --- | --- | --- |
|  | Mean | SD | Mean | SD |
| **Good collaboration and teamwork** | 1.915*** | -0.592** | 1.104*** | -0.008 |
| *(Ref: Poor collaboration and teamwork)* | [0.145] | [0.263] | [0.036] | [0.204] |
| **Supportive and accessible managers** | 1.246*** | 0.499*** | 0.770*** | 0.002 |
| *(Ref: Unsupportive and inaccessible managers)* | [0.088] | [0.124] | [0.022] | [0.095] |
| **Low work pressure** | 1.162*** | 0.738*** | 0.717*** | 0.474*** |
| *(Ref: High work pressure)* | [0.097] | [0.111] | [0.026] | [0.036] |
| **Good opportunities for career development** | 0.832*** | 0.716*** | 0.678*** | 0.431*** |
| *(Ref: Limited opportunities for career development)* | [0.068] | [0.103] | [0.020] | [0.038] |
| **Flexible working patterns** | 0.954*** | 0.686*** | 0.533*** | 0.071 |
| *(Ref: Lack of flexible working patterns)* | [0.075] | [0.110] | [0.018] | [0.178] |
| **Good recognition, skills are valued** | 0.817*** | 0.347 | 0.543*** | 0.104 |
| *(Ref: Low recognition, skills are not valued)* | [0.067] | [0.234] | [0.018] | [0.119] |
| **Good organisational support, well structured** | 0.480*** | 0.560*** | 0.454*** | 0.333*** |
| *(Ref: Lack of organisational support, poorly structured)* | [0.062] | [0.114] | [0.019] | [0.045] |
| **Salary** | 0.054*** |  | 0.040*** |  |
|  | [0.003] |  | [0.001] |  |
| Constant (Job choice A) | 0.133*** | -0.005 | 0.203*** | 0.002 |
|  | [0.050] | [0.127] | [0.017] | [0.071] |
| Observations | 10,020 |  | 56,496 |  |
| Number of respondents | 835 |  | 4708 |  |
| *** p<0.01, ** p<0.05 |  |  |  |  |
|  | WTP (%) | Annual average income (£) | WTP (%) | Annual average income (£) |
| Good collaboration and teamwork | -35.5% | £12,510.5 | -27.6% | £9,101.9 |
| Supportive and accessible managers | -23.1% | £8,140.0 | -19.3% | £6,348.2 |
| Low work pressure | -21.5% | £7,591.2 | -17.9% | £5,911.3 |
| Good opportunities for career development | -15.4% | £5,435.4 | -17.0% | £5,589.8 |
| Flexible working patterns | -17.7% | £6,232.4 | -13.3% | £4,394.3 |
| Good recognition, skills are valued | -15.1% | £5,337.4 | -13.6% | £4,476.7 |
| Good organisational support, well structured | -8.9% | £3,135.8 | -11.4% | £3,743.0 |
